# Double minute amplification of mutant PDGF receptor α in a mouse glioma model

**DOI:** 10.1038/srep08468

**Published:** 2015-02-16

**Authors:** Hongyan Zou, Rui Feng, Yong Huang, Joseph Tripodi, Vesna Najfeld, Nadejda M. Tsankova, Maryam Jahanshahi, Lorin E. Olson, Philippe Soriano, Roland H. Friedel

**Affiliations:** 1 Fishberg Department of Neuroscience, Friedman Brain Institute; 2 Department of Neurosurgery, Icahn School of Medicine at Mount Sinai, New York, NY 10029; 3 Tumor Cytogenomics Laboratory, Icahn School of Medicine at Mount Sinai, New York, NY 10029; 4 Department of Pathology, Icahn School of Medicine at Mount Sinai, New York, NY 10029; 5 Department of Developmental and Regenerative Biology, Icahn School of Medicine at Mount Sinai, New York, NY 10029; 6 Department of Oncological Sciences, Icahn School of Medicine at Mount Sinai, New York, NY 10029

## Abstract

In primary brain tumors, oncogenes are frequently amplified and maintained on extrachromosomal DNA as double minutes (DM), but the underlying mechanisms remain poorly understood. We have generated a mouse model of malignant glioma based on knock-in of a mutant PDGF receptor α (PDGFRα) that is expressed in oligodendrocyte precursor cells (OPCs) after activation by a Cre recombinase. In the tumor suppressor INK4/Arf^−/−^ background, mutant animals frequently developed brain tumors resembling anaplastic human gliomas (WHO grade III). Besides brain tumors, most animals also developed aggressive fibrosarcomas, likely triggered by Cre activation of mutant PDGFRα in fibroblastic cell lineages. Importantly, in the brain tumors and cell lines derived from brain tumor tissues, we identified a high prevalence of DM *Pdgfra* gene amplification, suggesting its occurrence as an early mutational event contributing to the malignant transformation of OPCs. Amplicons extended beyond the *Pdgfra* locus and included in some cases neighboring genes *Kit* and *Kdr*. Our genetically defined mouse brain tumor model therefore supports OPC as a cell of origin for malignant glioma and offers an example of a defined temporal sequence of mutational events, thus providing an entry point for a mechanistic understanding of DM gene amplification and its functionality in gliomagenesis.

Focal amplification of genomic DNA is a pathological hallmark in many solid tumors, including glioblastoma, the most frequent and deadly form of primary brain cancer^1^. Amplicons typically comprise 0.5–10 MB of DNA and exist either as circular extrachromosomal structures termed double minutes (DM), or as intrachromosomal concatenated repeats termed homogenously staining regions (HSR)^2^. These two forms of gene amplification are interrelated and possibly interconvertable^3^. Several models have been proposed on how DM arise during chromosome replication, including replication fork stalling, fork collision, and fragmentation of larger chromosomal segments^3^,^4^,^5^,^6^. However, mechanistic details of DM formation and its functionality in cancer development remain poorly understood. For instance, despite high prevalence in glioblastoma^2^, it is unclear whether DM gene amplification occurs as an early mutational event contributing to malignant transformation, or at a later stage in advanced glioblastomas as a result of global genomic instability^7^,^8^.

Increased activity of the receptor tyrosine kinase (RTK) PDGF receptor α (PDGFRα) is frequently encountered in glioblastoma and commonly associated with amplification of the *PDGFRA* gene^9^,^10^,^11^,^12^,^13^,^14^. Additionally, *PDGFRA* amplification is often associated with point mutations or structural variants that are thought to increase the intensity of PDGFRα signaling (Refs. 14, 15; http://www.cbioportal.org). A number of mouse glioma models have been generated by overexpressing secreted PDGF ligands in the brain (for review, see Refs. 16, 17), but since secreted PDGF ligands may act through both autocrine and paracrine mechanisms, the cell of origin cannot be conclusively defined in these glioma models. These models also do not address the prevalence of *PDGFRA* gene amplification and its oncogenic role in gliomagenesis. Providing an adequate animal model to investigate these fundamental questions is central to advancing glioma research and therapeutic options.

We have developed a new glioma mouse model based on cell-autonomous activation of PDGFRα in oligodendrocyte precursor cells (OPCs). Specifically, we utilized a conditional knock-in of a PDGFRα point mutation that reduces auto-inhibition of the kinase domain^18^. The knock-in design ensures that the expression of mutant PDGFRα is under the control of the endogenous *Pdgfra* promoter, which, in the CNS, is active in OPCs^19^. When bred on INK4/Arf^−/−^ background, a common tumor suppressor deletion in glioblastoma, mice developed spontaneous primary brain tumors between 15–30 weeks of age. Brain tumors displayed pathological characteristics of human high grade gliomas (WHO grade III), thus providing direct evidence for OPC as a cell of origin for malignant glioma. Importantly, we identified a high prevalence of amplification of the *Pdgfra* mutant allele as DM in not only advanced, but also early stage grade III gliomas, thus supporting RTK amplification as an important early event in the malignant transformation of OPCs. In summary, our study describes a novel glioma model that provides an example of a defined temporal sequence of mutational events in the malignant transformation of OPCs, starting from an activating RTK mutation and loss of a tumor suppressor, followed by amplification of the mutant RTK in the form of DM.

## Results

### A glioma mouse model driven by PDGFRα overactivity

**Figure 1:**
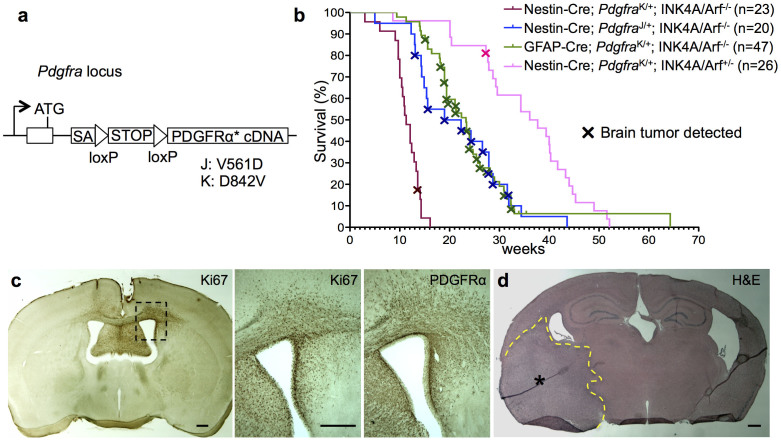
PDGFRα driven brain tumor model. (a) Schematic diagram of PDGFRα J/K knock-in alleles. ATG, start codon; SA, splice acceptor; STOP, PGK-neo cassette. (b) Kaplan-Meier survival curves of 4 mouse mutant cohorts with brain tumors. Mice generally succumbed to subcutaneous fibrosarcomas, and brain tumors were detected by histological analysis. (c) Example of early stage tumor growth, as revealed by IHC for proliferation marker Ki67 and PDGFRα. Note high density of Ki67+ proliferating cells in tumor area, increased expression level of PDGFRα, and invasive migration of tumor cells through corpus callosum into contralateral hemisphere. (d) H&E staining of an advanced brain tumor growth (asterisk in tumor centre, dashed line demarcates expansion). Scale bars: 50 μm (c, d).

To generate a glioma mouse model based on cell-autonomous activation of PDGFRα in OPCs, we utilized mice that carry conditional knock-in alleles of PDGFRα with the point mutations V561D (termed J for “juxtamembrane”) or D842V (termed K for “kinase domain”)^18^,^20^ (Fig. 1a). Both point mutations confer kinase overactivity due to reduced autoinhibition, with the K mutation being to some extent a stronger activator than the J mutation^18^. The PDGFRα J or K mutant alleles are expressed from the endogenous *Pdgfra* promoter after Cre recombinase-mediated excision of a STOP cassette. We crossed the conditional mutant mice to Nestin-Cre or GFAP-Cre transgenic lines, leading to Cre activation of mutant PDGFRα in all forebrain areas (Fig. S1). The knock-in design restricts in the CNS PDGFRα J/K expression to OPCs, which endogenously express PDGFRα^19^,^21^. Animals with overactive PDGFRα did not exhibit higher numbers of OPCs or mature oligodendrocytes (Fig. S2a). Furthermore, OPCs isolated from mutants proliferated at a normal rate in vitro (Fig. S2b) and mutant cohorts developed no signs of brain tumors, indicating that PDGFRα J/K mutation by itself is insufficient to increase OPC proliferation or drive gliomagenesis.

To facilitate the incidence of brain tumors, we bred PDGFRα J/K mutant mice onto the background of INK4A/Arf*^−/−^*, a frequent tumor suppressor mutation in glioblastoma^1^. Mice bearing both PDGFRα J/K and INK4A/Arf^−/−^ mutations developed normally until early adulthood and displayed normal numbers of OPC (Fig. S3a, b). Interestingly, at ages of 15–30 weeks, an estimated 10% of the animals in the GFAP-Cre; Pdgfrα^K/+^; INK4A/Arf^−/−^ cohort displayed an enlarged skull and apparent neurological symptoms (Fig. S3c), indicative of advanced primary brain tumors (see below). It is noteworthy that about 90% of mice in this GFAP-Cre activated cohort and 100% in the Nestin-Cre; Pdgfrα^K/+ or J/+^; INK4A/Arf^−/−^ cohort were affected by aggressive subcutaneous fibrosarcomas (Fig. S3c), presumably derived from Nestin-Cre or GFAP-Cre activation of PDGFRα J/K in fibroblastic cell lineages in the skin (Fig. S1a). The histological characteristics of the fibrosarcomas, including overexpression of *Pdgfra* and high grade features such as pleomorphic nuclei and numerous mitotic figures, were virtually identical to tumors that were previously described in detail in a Pdgfrα^K/+^; INK4A/Arf^−/−^ tumor model with general Cre activation^18^. Consequently, most cohort mice in our study had to be sacrificed due to fast growing fibrosarcomas before brain tumors could reach larger sizes, and our subsequent studies were mostly conducted on gliomas at relatively early stages of brain tumor growth.

Sacrificed mice were screened for signs of neoplastic growth in brains by immunostaining for PDGFRα and proliferation marker Ki67 (see Fig. 1b for survival curves and brain tumor incidence). More than 50% of animals in the mutant cohorts GFAP-Cre; Pdgfra^K/+^; INK4A/Arf^−/−^ and Nestin-Cre; Pdgfra^J/+^; INK4A/Arf^−/−^ harbored highly proliferative areas in the brain, reflecting early (Fig. 1c and S4a) and occasionally more advanced stages of brain tumor growth (Fig. 1d; Fig. S4b). In the Nestin-Cre; *Pdgfra*^K/+^; INK4A/Arf^−/−^ cohort, mice generally succumbed to fibrosarcomas by 15 weeks, before brain tumors were detectable (Fig. S3a). In the background of heterozygous INK4A/Arf deletion (Nestin-Cre; *Pdgfra*^K/+^; INK4A/Arf^+/−^), animals developed brain tumors only rarely, and cohort mice with heterozygous INK4A/Arf mutation alone (INK4A/Arf^+/−^) never developed brain tumors (Fig. S3a). Various regions of the forebrain were affected by tumor growth, including cortex, striatum, and thalamus (Fig. 1c, d; Fig. S4). All brain tumors displayed a highly invasive phenotype, with preferential migration routes along fiber tracts in the corpus callosum (Fig. 1c). We also observed a consistent molecular feature of increased immunointensity for PDGFRα in brain tumor cells as compared to normal OPCs (Fig. 1c; Fig. S4b), suggesting that not only the kinase overactivity from the J/K point mutation, but also upregulation of PDGFRα expression are required for the malignant transformation of OPCs.

### PDGFRα-driven murine brain tumors resemble human grade III gliomas

**Figure 2:**
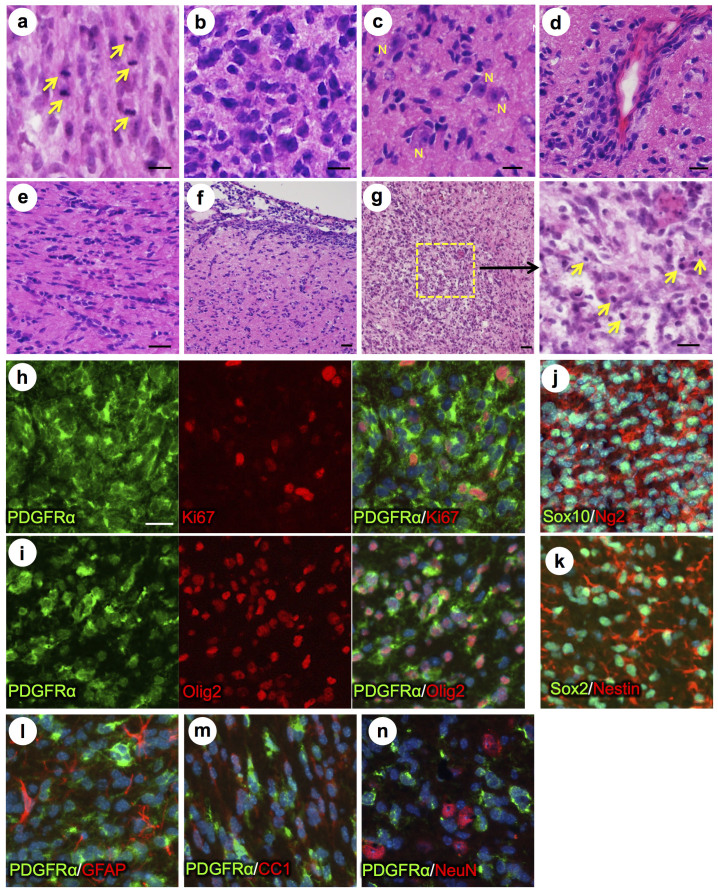
PDGFRα driven brain tumors display features of high grade glioma. (a–g) Histopathological analysis of tumor areas by H&E staining shows a high concentration of mitotic figures (a, arrows), high cellularity and nuclear atypia (b), perineuronal satellitosis (c; N, neuronal nuclei), perivascular growth (d), intrafascicular growth (e), subarachnoid spreading (f), and areas of incipient necrosis (g; arrows point to pyknotic nuclei). (h–k) IF labeling of brain tumor sections for cell type specific markers. Nuclei labeled with DAPI are shown in blue. Tumor cells with high PDGFRα expression were highly proliferative, as seen by proliferation marker Ki67 (h), and express the OPC cell lineage markers Olig2, Sox2, Sox10, and Ng2, as well as the neural stem cell marker Nestin (i–k). Tumor cells were negative for immunosignal of astroglial marker GFAP, mature oligodendrocyte marker APC-CC1, and neuronal marker NeuN (l–n). Scale bars: 10 μm (a–g), 20 μm (h–n).

We analyzed brain tumors histologically by H&E staining, which revealed densely cellular and diffusely infiltrating glial neoplasms, with several histopathological features of anaplastic gliomas (WHO grade III): brisk mitotic activity, hypercellularity, cellular pleomorphism, and marked nuclear atypia, i.e. hyperchromatic nuclei with irregular contours (Fig. 2a, b). Tumor cells were often arranged in secondary structures of Scherer, i.e. perineuronal satellitosis, perivascular cuffing, intrafascicular growth, and subarachnoid spreading (Fig. 2c–f). At the time of sacrifice, most brain tumors had not reached a size at which necrotic areas became widespread; however, we detected areas of decreased cellularity, rarefaction of neuropil, presence of pyknotic nuclei and apoptotic debris, likely representing incipient necrosis (Fig. 2g). Based on cellularity and morphological appearances, virtually all tumors were comparable with human high grade gliomas (WHO grade III), such as anaplastic oligodendrogliomas or anaplastic astrocytomas, but lacked pseudopalisading necrosis or glomeruloid microvascular proliferation that are diagnostic of WHO grade IV glioblastoma.

Further analysis by immunofluorescence revealed several consistent molecular features, such as increased PDGFRα expression, high proliferative index by Ki67 staining (Fig. 2h), and expression of OPC markers Olig2, Sox10, Ng2, and Sox2 (Fig. 2i–k). Additionally, tumor cells express Nestin, a neural stem cell (NSC) marker that is activated during malignant transformation of OPC^22^ (Fig. 2k). In contrast, markers of differentiated neural cells, such as GFAP (for astrocytes), APC-CC1 (for mature oligodendrocytes), and NeuN (for neurons), were not detectable in brain tumor cells (Fig. 2l–n).

### Amplification of PDGFRα K allele in glioma cells

For further analysis on a cellular level, we derived cell lines from forebrain tissue of mutant and control mice and cultured them in neural stem cell media under spherogenic conditions, which allows for selection of the so-called glioma stem cells (GSC; also known as tumor-propagating cells). Cell lines derived from advanced brain tumors are expected to consist predominantly of GSCs, whereas cell lines from wild-type brain will only consist of normal neural progenitors. Cell lines derived from tissue of early stage gliomas with lower numbers of tumor cells may consist of either GSCs, normal neural progenitors, or both cell types.

**Figure 3:**
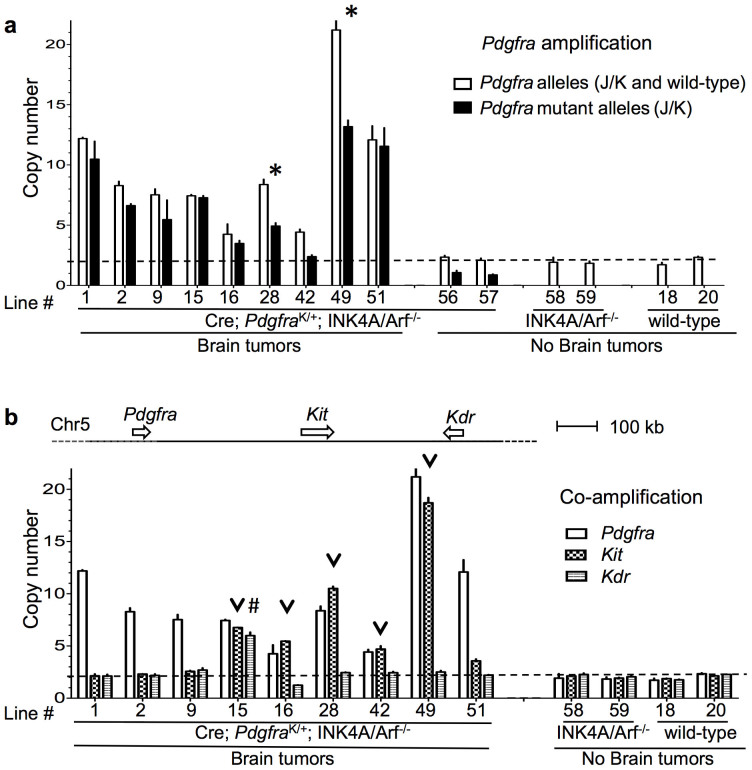
Gene amplification of PDGFRα in glioma cell lines. (a) Increased *Pdgfra* allele copy numbers detected by qPCR in 9 cell lines derived from glioma-bearing brains, but not in control cell lines. PCR primers were either generic for all *Pdgfra* alleles or specific for *Pdgfra* J/K mutant alleles. Asterisks denote cell lines in which both mutant and wild-type allele amplifications have occurred. (b) Amplification can also include the adjacent genes *Kit* (co-amplified in 5 out of 9 lines) and *Kdr* (in 1 out of 9 lines). Arrowheads or # signs denote co-amplification of *Kit* or *Kdr*.

We established cell lines from mice in our brain tumor cohorts, including 5 mice with advanced grade III gliomas that had resulted in a bulging skull and behavioral impairment, and 10 mice with early stage grade III gliomas detected only by histological analysis. To investigate the mechanism of the upregulation of PDGFRα expression in glioma cells, we screened these cell lines for *Pdgfra* gene copy numbers by qPCR. We observed *Pdgfra* amplification in 9 of the 15 lines derived from tumor-bearing mice, including all 5 lines from mice bearing advanced gliomas, and 4 lines from mice bearing early stage gliomas (Fig. 3a). The average copy number of the *Pdgfra* gene in these 9 glioma cell lines varied from 4 to 21 copies (Fig. 3a). The other 6 lines from mice bearing early stage gliomas showed no *Pdgfra* gene amplification. It is worth noting that these 6 lines may consist predominantly of normal neural progenitors and not glioma cells; another possibility is that in these glioma cases alternative mechanisms, such as epigenetic modifications, transcription factor activation, or increased protein stability, might be responsible for the upregulation of PDGFRα expression. As controls, we also screened spherogenic cell lines derived from wild-type mice, INK4A/Arf^−/−^ mice, and brain tumor cohort mice with no detectable brain tumors, and observed no *Pdgfra* amplifications (Fig. 3a). Taken together, these data demonstrate that *Pdgfra* amplification is a frequent event that can occur during early stages of malignant glioma development and may serve as an important mechanism for the upregulation of PDGFRα expression in gliomas.

### Preferential amplification of the mutant allele of PDGFRα

To distinguish gene amplification of PDGFRα J/K mutant vs. wild-type allele, we utilized qPCR primers specific for the mutant alleles. In the majority of glioma cell lines tested (7 out of 9), the copy numbers of the mutant allele approached the total number of all *Pdgfra* alleles (note that mutant specific primers can not detect the wild-type *Pdgfra* allele), indicating a preferential amplification of the PDGFRα J/K mutant allele (Fig. 3a). In the remaining two glioma lines (line #28 and #49), qPCR results showed that both mutant and wild-type alleles were amplified (Fig. 3a).

To determine if the amplification is focally restricted to the *Pdgfra* gene, or encompasses a larger chromosomal area, we first probed for amplicons 50 kb upstream and downstream of the *Pdgfra* locus. Of the 6 cell lines tested, 5 lines showed amplification extending beyond the *Pdgfra* locus by at least 50 kb in both directions, and one line showed amplification only downstream of the *Pdgfra* locus, indicating that amplicons are in general larger than 100 kb (Fig. S5a). Next, we investigated copy numbers of two adjacent RTK genes, *Kit* and *Kdr*, located approximately 0.4 Mb and 0.8 Mb downstream of *Pdgfra*, respectively. Of note, *Kit* and *Kdr* are frequently co-amplified with *Pdgfra* in human glioblastoma^23^. We found that among the 9 *Pdgfra*-amplified glioma cell lines tested, *Kit* was co-amplified in 5 lines, and one of these lines also had additional *Kdr* co-amplification (Fig. 3b). Finally, we analyzed amplification of *Mdm2* and *Egfr*, two of the most commonly amplified oncogenes in glioblastoma^1^, but located on different chromosomes than *Pdgfra*. We found only two copies of each gene in all cell lines tested (Fig. S5b), suggesting absence of widespread gene amplification or global genomic instability in this tumor model.

### Amplified PDGFRα is maintained as extrachromosomal DM

**Figure 4:**
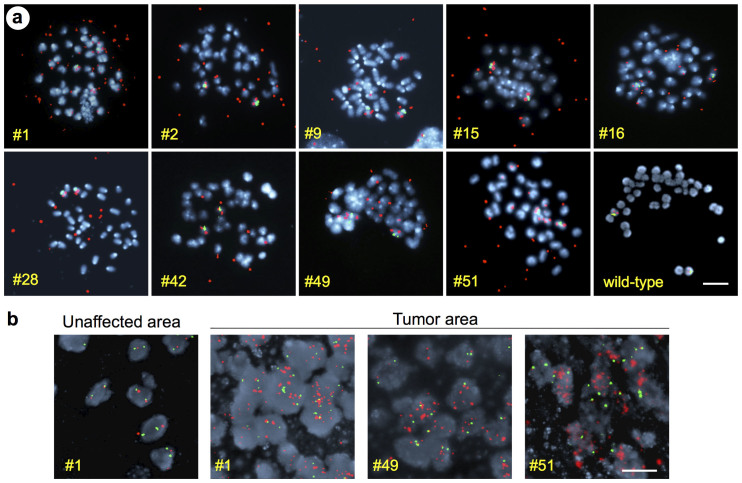
Double minute amplification of PDGFRα. (a) Metaphase spreads of 9 *Pdgfra* amplified cell lines and one wild-type control line, hybridized with FISH probe for PDGFRα locus (red) and chromosome 5 centromere region (green), revealing amplification of PDGFRα locus as extrachromosomal DM. The cell line number is indicated in each image. (b) FISH hybridization on brain tumor sections confirms amplification of PDGFRα locus in situ. Examples of three brain tumors and one unaffected adjacent area are shown. The number of the corresponding cell line is indicated in each image. Chromosomes and nuclei labeled with DAPI are shown in blue. Scale bars: 5 μm (a), 20 μm (b).

To determine the cytogenetic nature of the *Pdgfra* amplification, we conducted fluorescent in situ hybridization (FISH) on metaphase spreads obtained from glioma cell lines using probes for the *Pdgfra* locus (located on chromosome 5) and for the centromere of chromosome 5 (internal control). In all *Pdgfra-*amplified glioma cell lines, we found that in addition to the two *Pdgfra* loci on the chromosome 5 pair, FISH signals were also abundant on small extrachromosomal pieces of DNA (Fig. 4a), corresponding to DM, a class of circular extrachromosomal DNA fragments. We did not detect *Pdgfra* amplicons integrated into chromosomes. The majority of DM that were visualized by DAPI counterstaining were positive for the *Pdgfra* FISH signal, but a few DM without *Pdgfra* amplicons were also detected (Fig. S6).

The *Pdgfra* copy numbers in individual cells from the same glioma cell line were highly variable (Fig. S7), likely reflecting unequal DM distribution among daughter cells during cell division^24^. It also highlights intratumoral heterogeneity of *Pdgfra* copy number status. We also screened directly sections of brain tumor tissues for *Pdgfra* amplification by FISH, and confirmed widespread presence of multiple *Pdgfra* amplicons in tumor areas (Fig. 4b), thus supporting the notion that *Pdgfra* amplification is a driving factor during gliomagenesis.

## Discussion

High grade gliomas are thought to be a heterogeneous form of cancer, originating from different neural cell types, including astrocytes, neural stem cells, and OPCs^25^,^26^. In our study, by activating a mutant PDGFRα cell-autonomously in the OPC population, we provide direct evidence for OPC as a cell of origin for malignant glioma. A series of previous studies have utilized overexpression of PDGF-A or -B in the mouse brain to induce gliomagenesis (reviewed in Refs. 16, 17). Of particular interest are studies that directed PDGF-B overexpression specifically to late OPCs/early oligodendrocytes, which strongly support OPC as cell of origin of glioma^27^,^28^. However, due to potential paracrine actions of secreted PDGF ligands on different cell types, the cell of origin could not be unambiguously determined. In contrast, our glioma model is based on expression of a mutant PDGFRα receptor under the control of the endogenous *Pdgfra* promoter in the OPC population^19^,^21^,^29^. Of note, expression of PDGFRα has also been reported in a population of neurovascular cells^21^,^30^, but the molecular features of gliomas observed in our study make it unlikely that this cell type contributed to tumorigenesis. Expression of PDGFRα in neural stem cells in the SVZ, as has been proposed earlier, was not detected in the present study, in agreement with more recent observations^29^,^31^.

The expression of mutant PDGFRα J/K in OPC by itself did not lead to gliomagenesis, but when combined with loss of INK4A/Arf, was able to drive gliomagenesis. This result is reminiscent of studies with mutant EGF receptor, which by itself triggers gliomagenesis only at very low levels and requires additional deletion of INK4A/Arf for frequent glioma formation^32^,^33^. These studies therefore suggest that loss-of-function mutations of INK4A/Arf or other tumor suppressor genes may be an early permissive event that facilitates transforming gene amplifications. A significant finding of our study is the spontaneous DM amplification of *Pdgfra*, preferentially of the mutant allele, in early stage gliomas. Hence, our model provides an example where two preexisting mutations–activating RTK mutation and INK4A/Arf deletion–facilitate tumorigenesis through a third mutational event, that is, amplification of the mutant RTK.

In human primary glioblastomas, which arise de novo, *PDGFRA* amplifications are found in 10–20% of cases, which makes *PDGFRA* the second most frequently amplified RTK gene in glioblastoma after *EGFR*^34^,^35^. Among primary glioblastomas of the proneural non-CpG island methylation (non G-CIMP) subtype, *PDGFRA* amplifications even reach a frequency of greater than 50%^1^. On the other hand, Secondary glioblastomas, which transform gradually from low grade G-CIMP gliomas, are characterized by expression of a proneural profile with high PDGF pathway activity^36^. Further studies will determine how well our glioma model of Pdgfra DM amplification is suited to study development of primary proneural glioblastomas, secondary glioblastomas, or both.

The challenge of studying gene amplification using patient-derived samples is that they invariably harbor numerous genetic mutations and display vast intratumoral heterogeneity, making it difficult to determine the conditions permissive for gene amplification. Intratumoral heterogeneity of gene amplification is further reflected by the fact that many glioblastomas carry DM amplifications of either *PDGFRA* or *EGFR* in intermingled tumor cell populations^23^,^34^,^35^. Our mouse glioma model provides a unique entry point for studying the propensity of glioblastoma to amplify RTKs, as our model is genetically defined with only two initiating mutations–a point mutant of PDGFRα with elevated kinase activity and loss of INK4A/Arf.

The high prevalence of *Pdgfra* amplification in our model suggests that *Pdgfra* amplification can occur as an early spontaneous event. This is in contrast to earlier concepts that gene amplification takes place in advanced tumors after accumulation of a large number of mutations that ultimately facilitate genomic amplifications^7^. In further support, *PDGFRA* amplification is present in more than 15% of human grade III anaplastic astrocytoma and anaplastic oligodendroglioma, consistent with *PDGFRA* amplification as an early transforming event during glioma progression^34^. The fact that we observed preferential amplification of the mutant but not the wild-type allele of *Pdgfra* implies that the kinase activity of the amplified PDGFRα is under positive selection during gliomagenesis. If *Pdgfra* amplification were simply a result of genomic instability, both the wild-type and the mutant alleles of *Pdgfra* would be amplified at similar frequencies.

Our approach of cultivating tumor cell lines allowed preparations of metaphase spreads that provided direct evidence for *Pdgfra* amplification as DM. We also confirmed that amplicons extend beyond the *Pdgfra* locus, resembling the situation in human glioblastoma^23^. Interestingly, in a mouse glioma model generated by loss of two tumor suppressors, PTEN and p53, spontaneous focal amplifications of RTK genes EGFR, MET, or PDGFRα as DM were described^37^. This study and ours demonstrate that loss of tumor suppressors such as Ink4A/Arf, or PTEN and p53, create a condition in which additional focal amplifications of RTKs can drive malignant transformation of glioma cells.

DM gene amplifications are numerically unstable in proliferating cell populations due to variable replication and uneven segregation between daughter cells. In our PDGFRα-driven glioma model, the copy numbers of PDGFRα vary among individual tumor cells. In the case of EGFR amplification in glioblastoma, mathematical modeling suggests that low level EGFR amplification is an early event, whereas high level amplification is a late event^8^. Recent modeling also suggests that a potential pathway to glioma encompasses initial PDGF-A overexpression followed by *PDGFRA* amplification at later stages^38^. Gene copy numbers in tumor cells are perhaps dictated by the need for an optimal tumor growth equilibrium, as excess gene copies may over-intensify signaling flux with detrimental consequences. For instance, tumor cells can dynamically reduce copy numbers of EGFR-vIII DM in response to tyrosine kinase inhibitor (TKI) treatment, but return back to previous levels upon TKI withdrawal^39^. Future studies will determine whether glioma cells under treatment with PDGFRα specific TKI exhibit a similar adaptive regulation of PDGFRα DM copies. Gene amplification by DM can also be associated with structural rearrangements of oncogenes^40^, hence further characterization of PDGFRα rearrangements will be needed to elucidate this mechanism. Obtaining a better mechanistic understanding of DM gene amplification and its oncogenic roles in the initiation and progression of glioma may lead to new therapeutic strategies for the treatment of brain tumors.

## Methods

### Mouse mutant alleles

PDGFRα J/K: *Pdgfra*^tm12Sor^ and *Pdgfra*^tm13Sor^ 18; Nestin-Cre: Tg(Nes-cre)1Kln^41^; GFAP-Cre: Tg(GFAP-cre)25Mes^42^; INK4A/Arf knockout: *Cdkn2a*^tm1Rdp^ 43; R26R-lacZ reporter: *Gt(ROSA)26Sor^tm1Sor^* 44. Brain tumor cohorts were bred on a mixed genetic background of FVB, C57BL/6J, and 124S4. Mice were euthanized in accordance with NIH Guidelines for the Care and Use of Laboratory Animals, and animal protocols were approved by the IACUC committee of Icahn School of Medicine at Mount Sinai.

### Histology

Antibodies and their dilutions used for immunostaining of brain sections: goat anti-PDGFRα (R&D Systems AF1062, 1:50); rabbit anti-PDGFRα (Santa Cruz Biotechnology SC-338, 1:50); rabbit anti-Ki67 (Abcam 15580, 1:500); rabbit anti-Olig2 (Millipore AB9610, 1:500); rabbit anti-Sox2 (Millipore AB5603, 1:200); goat anti-Sox10 (R&D Systems AF2864, 1:50); mouse anti-Nestin (Millipore MAB353, 1:250); rabbit anti-Ng2 (Millipore AB520, 1:500); rabbit anti-NeuN (Novus NBP1-77686SS, 1:200); rabbit anti-GFAP (Invitrogen 180063, 1:200); mouse anti-APC, clone CC1 (Calbiochem OP80, 1:20).

Secondary antibodies were coupled with Alexa fluorophores (Jackson Immunoresearch) or biotinylated for ABC signal amplification and peroxidase DAB staining (Vector Labs).

For mRNA in situ hybridization, cryosections were hybridized with Digoxigenin-labeled riboprobes, which were synthesised from the plasmids pBS-Pdgfra-ISH-1.6 kb and pCR4-Plp1-ISH-2.0 kb, and probe hybridization was detected with AP-conjugated anti-Dig Fab fragments (Roche) and NBT/BCIP staining.

Hematoxylin & Eosin staining was performed with Shandon Gill #2 Hematoxylin and Eosin-Y (Thermo Scientific).

### Glioma cell lines

The anterior third of the forebrain of mice was dissected and tissue was minced into small pieces with a scalpel blade and then incubated in Accutase (Invitrogen) for 15 min at 37°C. Cells were gently dissociated with a 5 ml pipette and the cell suspension was filtered through a 40 μm cell strainer (BD Falcon) and then cultured in Neural Stemcell media (Stemcell Technologies) for several days until spheres of >200 μm had formed^45^. Spheres were passaged by Accutase dissociation and archived in liquid nitrogen storage in Neural Stemcell media containing 10% DMSO.

### Oligodendroctyte precursor cell culture

Oligodendrocyte precursor cells (OPC) were isolated from cortices of P5 mice by Papain digest and selective immunopanning with anti-PDGFRα antibodies, and then cultured in poly-D-lysine coated tissue culture dishes with defined OPC media containing PDGF-A^46^.

### Fluorescence in situ hybridization (FISH)

Cell lines were cultivated over night in presence of 0.1 μg/ml colcemid (Invitrogen) before cytogenetic preparation by KCl treatment and fixation. The mouse BACs RPCI-23-222P8 and RPCI-23-413L22 were used as templates for synthesis of FISH probes for the chromosome 5 centromere and *Pdgfra* gene locus, respectively (Empire Genomics, Buffalo, NY). Cells and tissue sections were hybridized with FISH probes according to manufacturer's instructions (Empire Genomics) and counterstained with DAPI.

### Quantitative PCR

Gene copy numbers were determined by qPCR of genomic DNA from cell lines with a SYBR green reaction mix (Quanta Biosciences) on an ABI7900 device and normalized to a PCR for the *Foxp2* locus. Primer sequences (5′-3′): Pdgfra: GGGGAGAGTGAAGTGAGCTG and CATCCGTCTGAGTGTGGTTG; PdgfraJ/K mutant allele only: CAAACTCTTCGCGGTCTTTC and CGAAGTTATATTAAGGGTTCCG; Pdgfra 50 kb upstream: GATGCTGAGACACTCCTCTTG and GACTTGCTTAGTGCATCTGTATTG; Pdgfra 50 kb downstream: GATAGAGGCCCATGTCTTCTTAC and GCAGACTAACATGACAGGAGAA; Egfr: CCTGGAAGAGACCTGCATTATC and GTTAAACCCACTACTGAGACAGG; Kit: CAGCACATAGCCCAGGTAAA and CAACTCTTGCCGAGCTGATA; Kdr: CTCCATGACATAAGGCCTACAC and CTGCCACAAGCCTACTGATAA; Mdm2: GGAAGTCGATGGTTGGGAATAG and AGCTGACAGAGAATGATGCTAAA; Foxp2: AGCTCCTTTGCCTTCTCCACTCTT and AGGGCAATGAAGCCAGTCTGTACT.

### Statistical Analysis

Graphpad Prism 5 software was used to calculate Kaplan-Meier survival curves and bar graph analyses. Data are presented as mean ± SEM.

## Author Contributions

H.Z., L.E.O., P.S. and R.H.F. designed research; R.F., Y.H., J.T., M.J., L.E.O. and R.H.F. conducted experiments; H.Z., V.N., N.T., L.E.O., P.S. and R.H.F. analyzed data; H.Z. and R.H.F. wrote the paper. All authors read and approved the final manuscript.

## Supplementary Material

Supplementary Information Supplementary Info
