# Supplementary material for: Double minute amplification of mutant PDGF receptor α in a mouse glioma model
Source: Sci Rep. 2015 Feb 16;5:8468. doi: 10.1038/srep08468 (PMC4329559; doi:10.1038/srep08468)
Supplement: Supplementary Information — Supplementary Info [file srep08468-s1.pdf]

Supplementary Information

Double minute amplification of mutant PDGF receptor α in a mouse glioma model

Hongyan Zou, Rui Feng, Yong Huang, Joseph Tripodi, Vesna Najfeld, Nadejda Tsankova, Maryam Jahanshahi, Lorin E. Olson, Philippe Soriano, Roland H. Friedel

Contents:

Supplementary Figures 1-7

Supplementary Figure 1

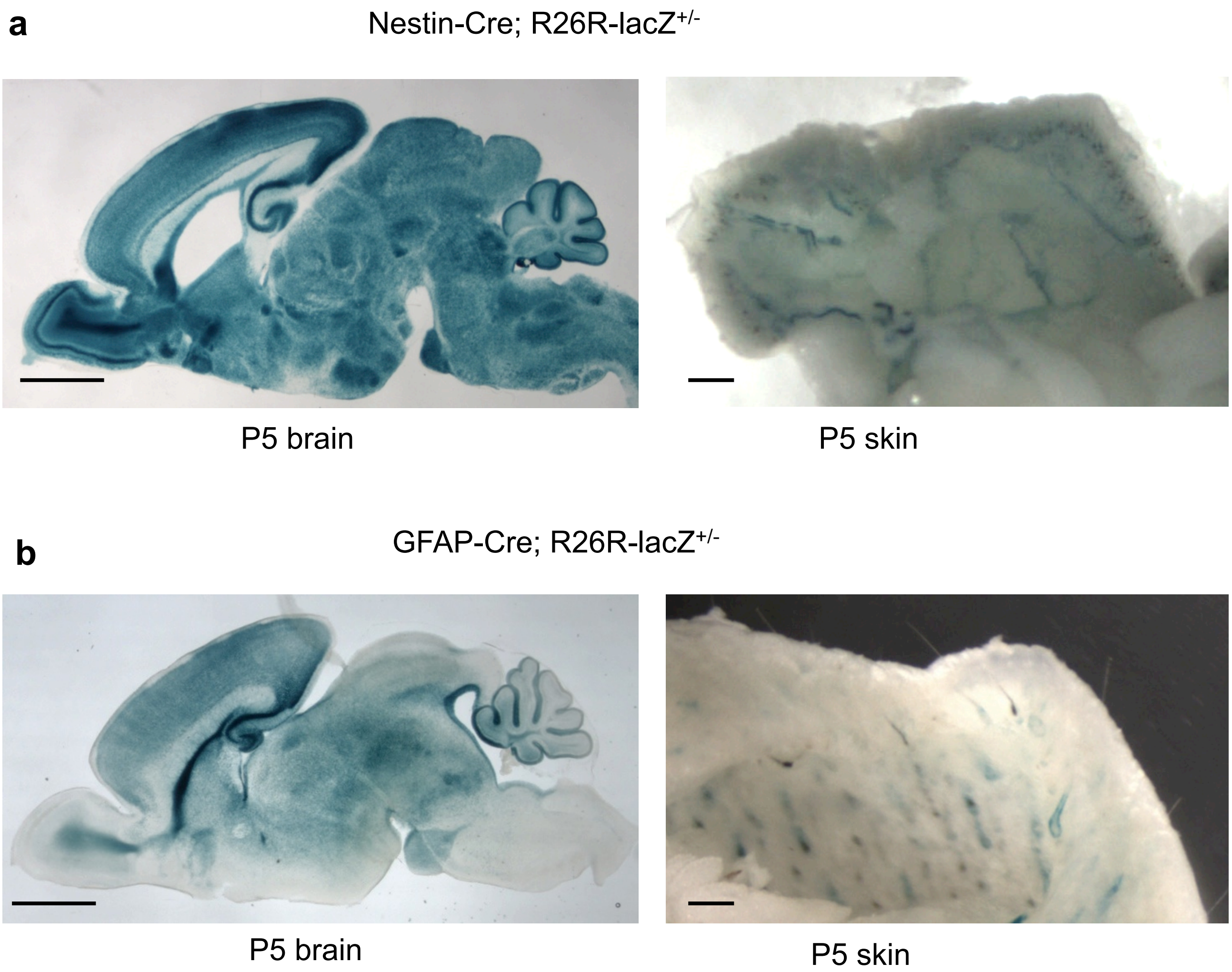

Figure S1. Comparison of Nestin-Cre and GFAP-Cre recombination lineages in brain and skin.
a, b) Nestin-Cre (a) or GFAP-Cre (b) mice were crossed with ROSA26R lacZ reporter mice, and X-Gal staining was performed to visualize Cre recombined cell lineages in doubly heterozygous offspring. On postnatal day 5 (P5), forebrains of both Nestin-Cre and GFAP-Cre mice were labeled, although the number of recombined cells was higher in Nestin-Cre than in GFAP-Cre mice. Recombined cell lineages were found in the skin of both Nestin- and GFAP-Cre mice at P5. Scale bars: 1 mm (a, b, left panels), 200 µm (a, b, right panels).

Supplementary Figure 2

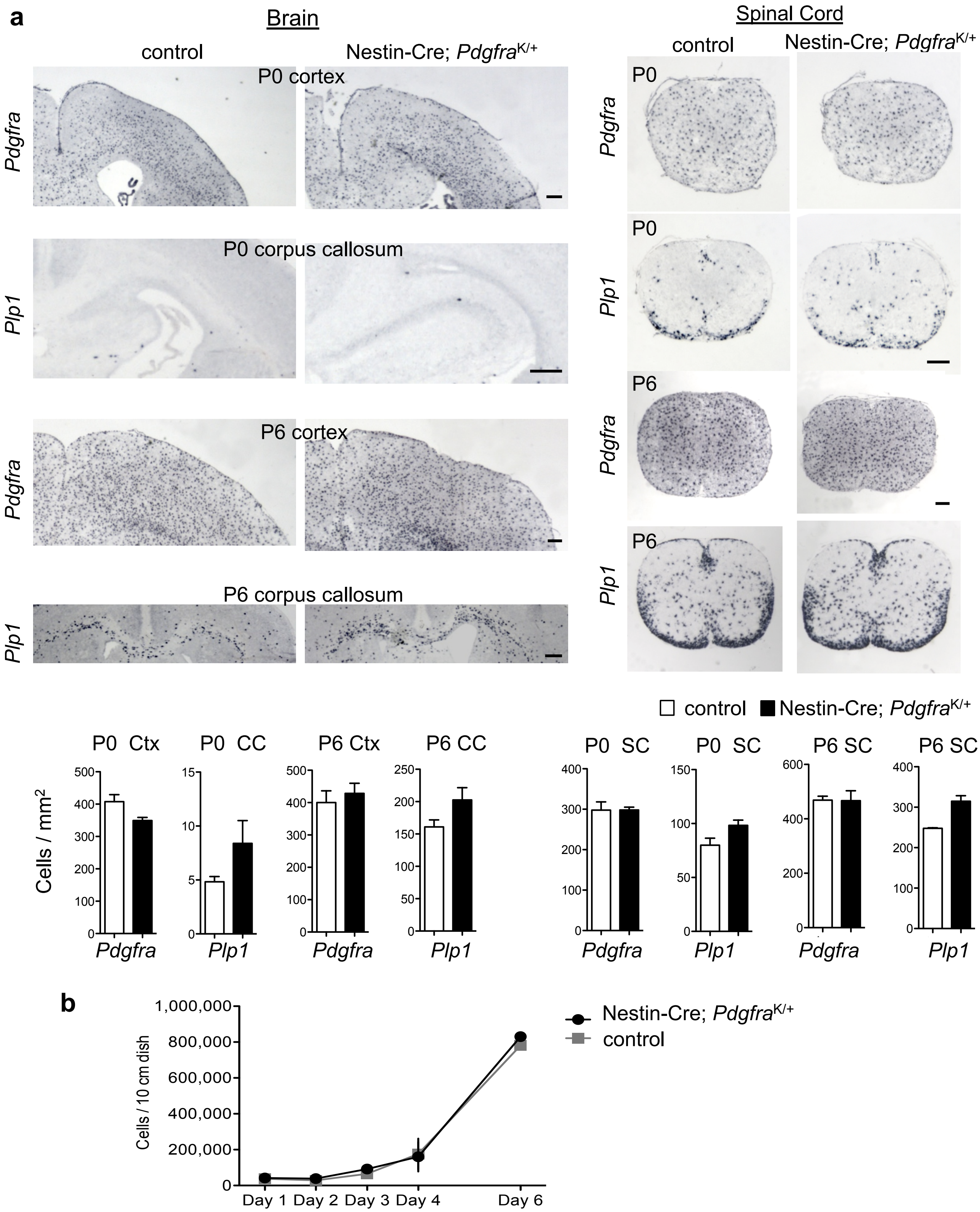

Figure S2. No effect of PDGFRα K activation on OPC expansion.

a) Activated PDGFRα K allele does not increase populations of OPCs or differentiated oligodendrocytes, as shown by in situ hybridizations for the OPC marker *Pdgfra* and the mature oligodendrocyte marker *Plp1*. P0 and P6 spinal cord tissue, cortex, and corpus callosum were analyzed. Quantification is shown below. Scale bars: 20 μm. b) Growth curve of OPCs isolated from Nestin-Cre; Pdgfra$^{K/+}$ animals or control littermates by immunopanning and cultivated for 6 days in PDGF-A containing OPC growth media. No difference in cell proliferation was detected.

Supplementary Figure 3

a

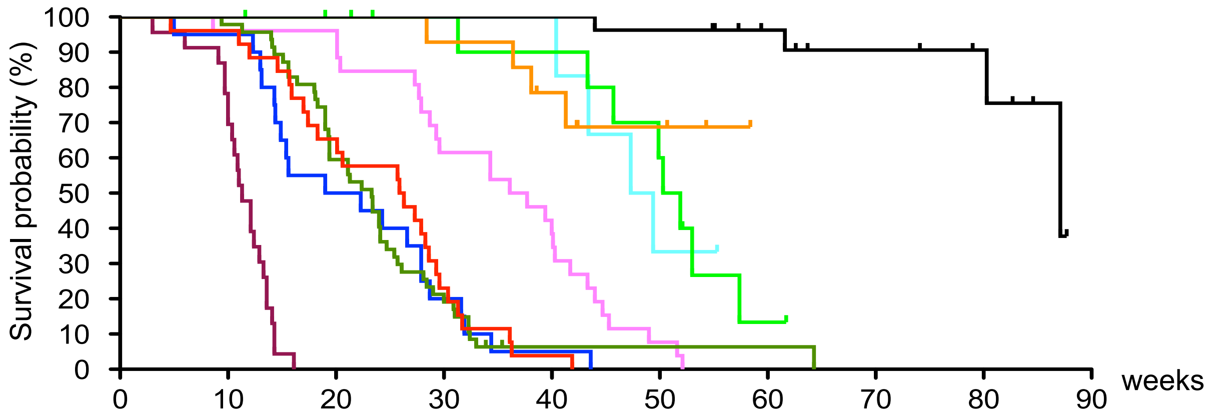

Cohort	n	Median survival (weeks)	Predominant cause of death	Brain tumor detected
Nestin-Cre; PDGFRα$^{K/+}$; INK4A/Arf$^{-/-}$	23	14.3	Fibrosarcoma	X
Nestin-Cre; PDGFRα$^{J/+}$; INK4A/Arf$^{-/-}$	20	20.6	Fibrosarcoma	X
GFAP-Cre; PDGFRα$^{K/+}$; INK4A/Arf$^{-/-}$	47	23.2	Fibrosarcoma	X
INK4A/Arf$^{-/-}$	26	26.1	Leukemia/Lymphoma	
Nestin-Cre; PDGFRα$^{K/+}$; INK4A/Arf$^{+/-}$	26	36.9	Fibrosarcoma	X
Nestin-Cre; PDGFRα$^{J/+}$; INK4A/Arf$^{+/-}$	6	48.3	Fibrosarcoma, Leukemia/Lymphoma	
GFAP-Cre; PDGFRα$^{K/+}$; INK4A/Arf$^{+/-}$	14	51.1	Fibrosarcoma, Leukemia/Lymphoma	
INK4A/Arf$^{+/-}$	14	>60	Leukemia/Lymphoma	
wild type	27	>90	n.d.	

b

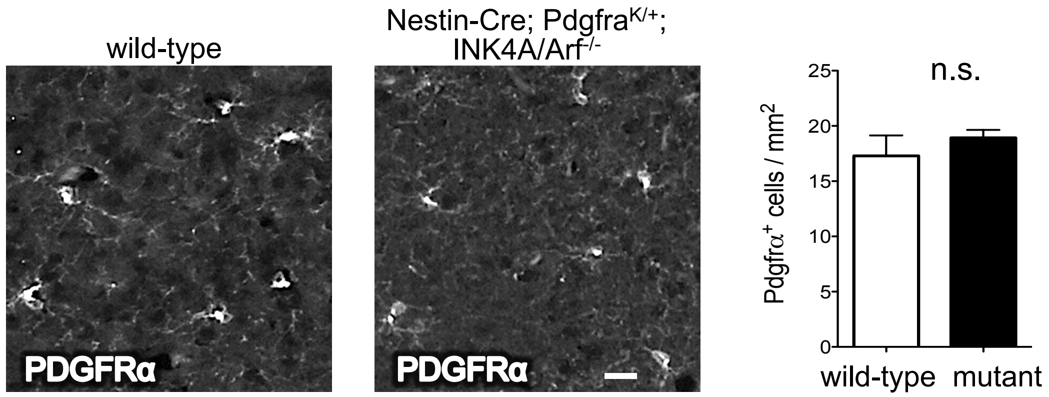

c

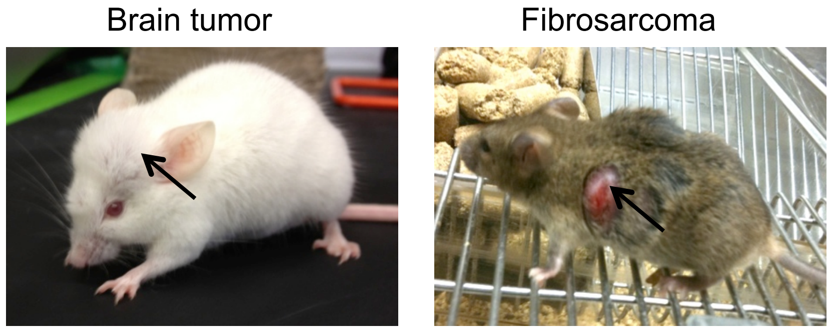

Figure S3. Brain tumor cohorts.

a) Kaplan-Meier survival curves of mouse cohorts with PDGFRα activation and/or INK4A/Arf deletion. In terms of mutagenicity, Nestin-Cre recombinase exhibited higher activity than GFAP-Cre, and PDGFRα K appeared to be a stronger mutant allele than J allele, leading to earlier tumor onset and shorter survival in the cohorts carrying the respective alleles. b) Animals in the Nestin-Cre; PDGFRα$^{K/+}$; INK4A/Arf$^{-/-}$ cohort, in which no brain tumor growth was detected, reveal no expansion of OPC population. Panels show immunofluorescence labeling and quantification for OPC marker PDGFRα in cortex of adult mice. Scale bar: 20 μm. c) Examples of mice affected by brain tumor with apparent expansion of cranium (left) or fibrosarcoma under skin with ulceration (right).

Supplementary Figure 4

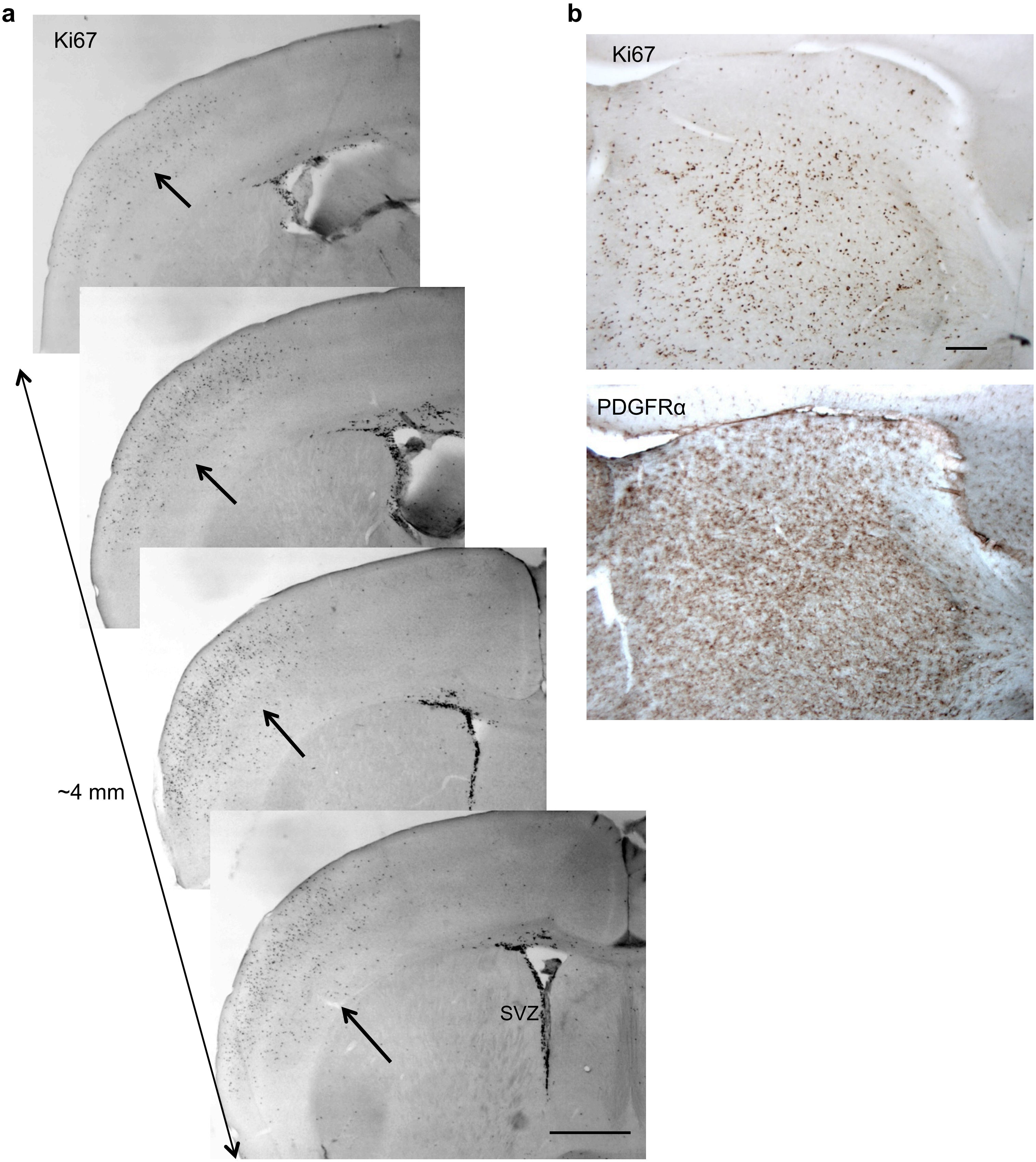

Figure S4. Examples of PDGFRα driven brain tumors.

a) Example of early brain tumor spreading diffusely through the cortex. Note that subventricular zone (SVZ) contains proliferating neural stem cells that also stain positive for the proliferation marker Ki67. b) Example of brain tumor that has infiltrated dorsal thalamus. Note increased PDGFRα expression in area that corresponds to the Ki67+ tumor area. Scale bars: 1 mm (a), 200 μm (b).

Supplementary Figure 5

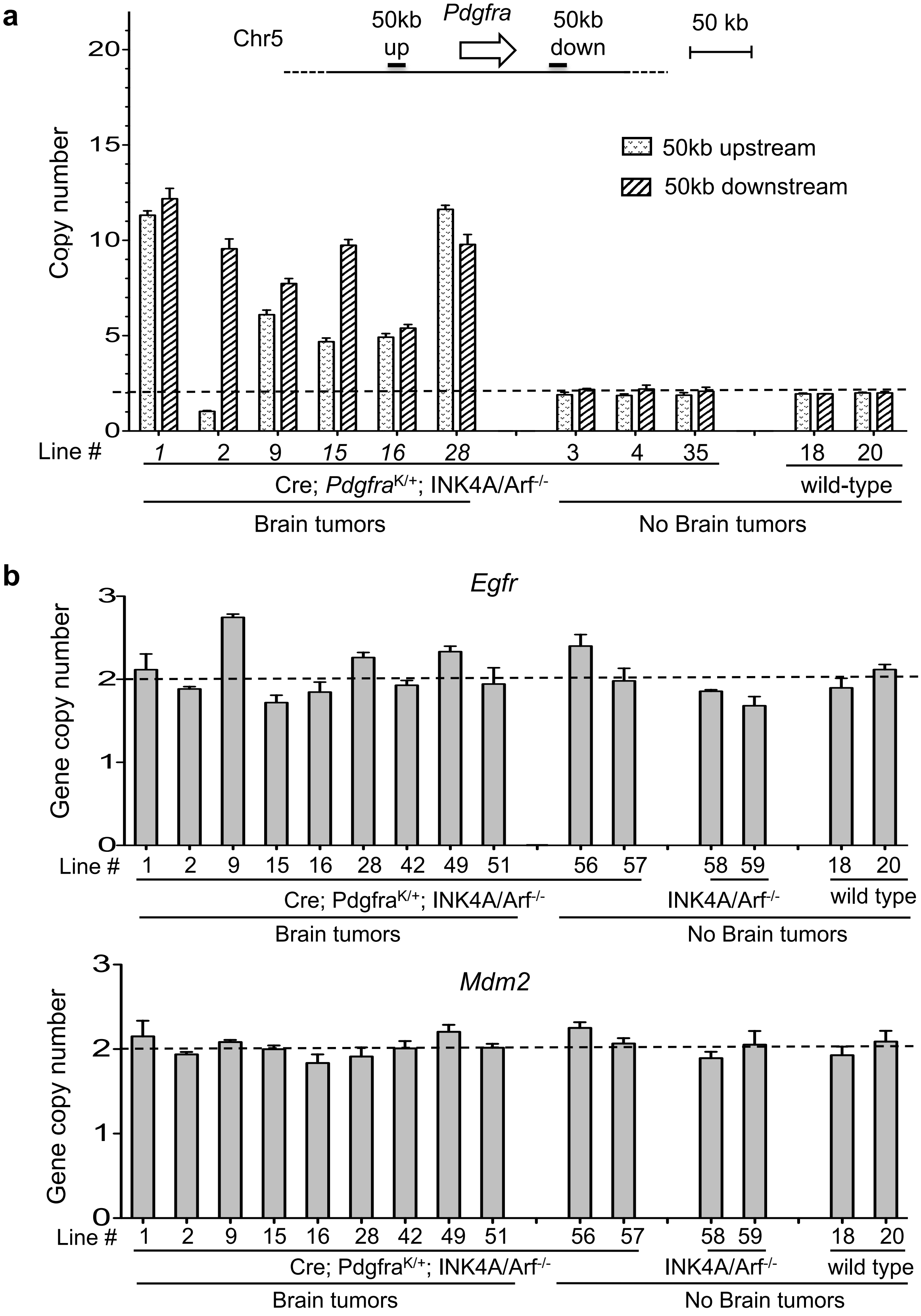

Figure S5. Amplification of genomic areas beyond the *Pdgfra* locus.
a) qPCR analysis of 6 glioma cell lines revealed that the *Pdgfra* amplicon contains also regions 50 kb upstream and downstream of the *Pdgfra* gene. Note that line #2 lacks amplification of the upstream area. b) qPCR analysis of glioma cell lines for the oncogenes *Egfr* and *Mdm2*, showed no gene amplification for these two loci in the PDGFRα mouse glioma model.

Supplementary Figure 6

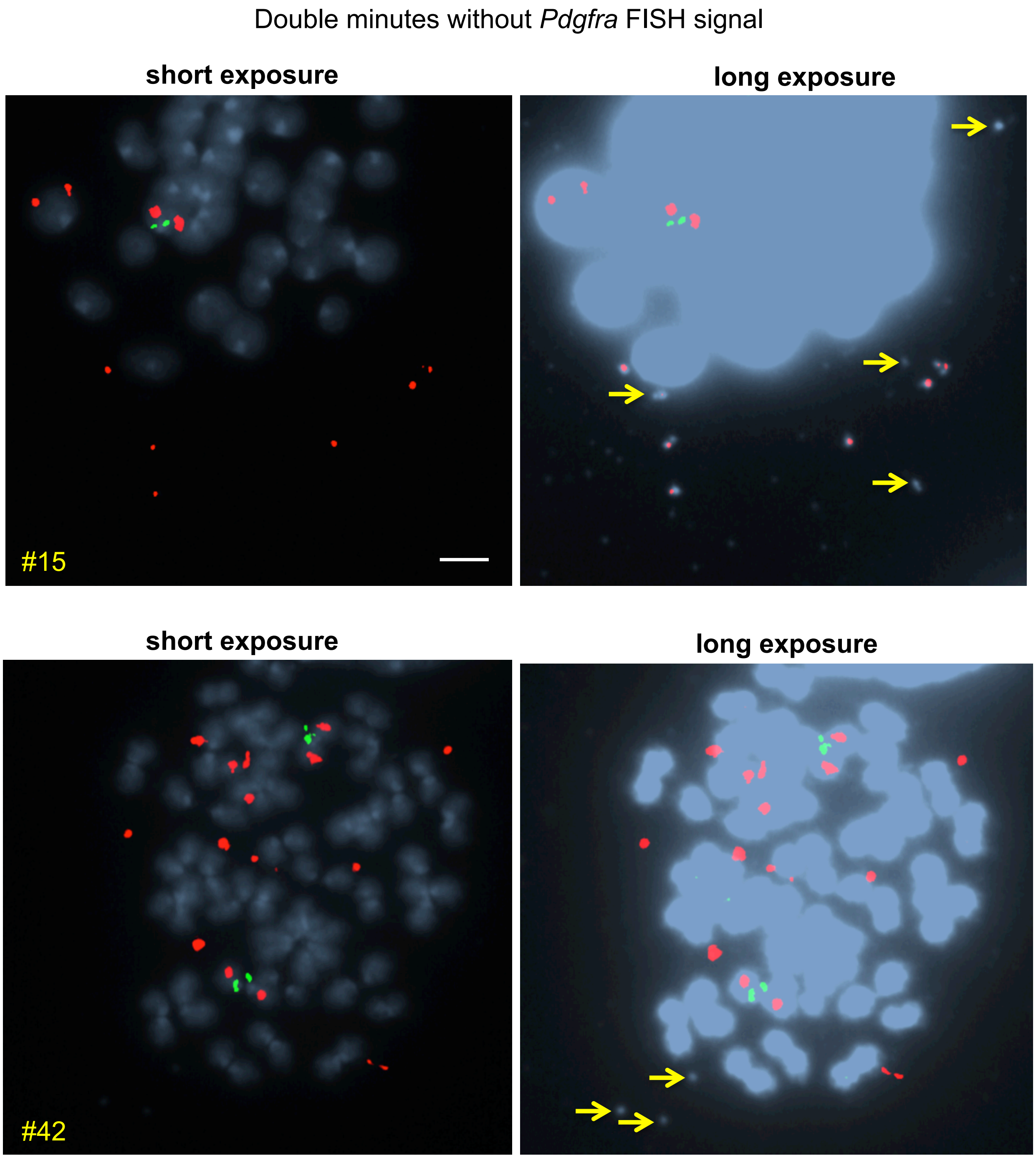

Figure S6. Glioma cells contain DM that are not *Pdgfra* positive.
Long exposure of DAPI stained metaphase preparations of glioma cell lines #15 and #42 reveals DM that do not carry *Pdgfra* amplicons (arrows). Scale bar: 2 µm.

Supplementary Figure 7

a

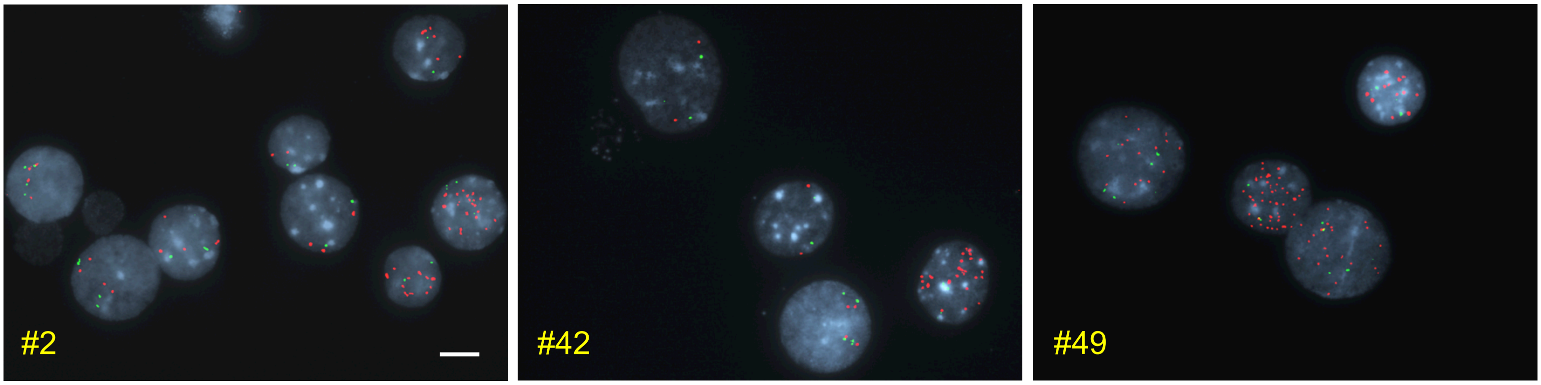

b

Distribution of *Pdgfra* copy number in glioma cells

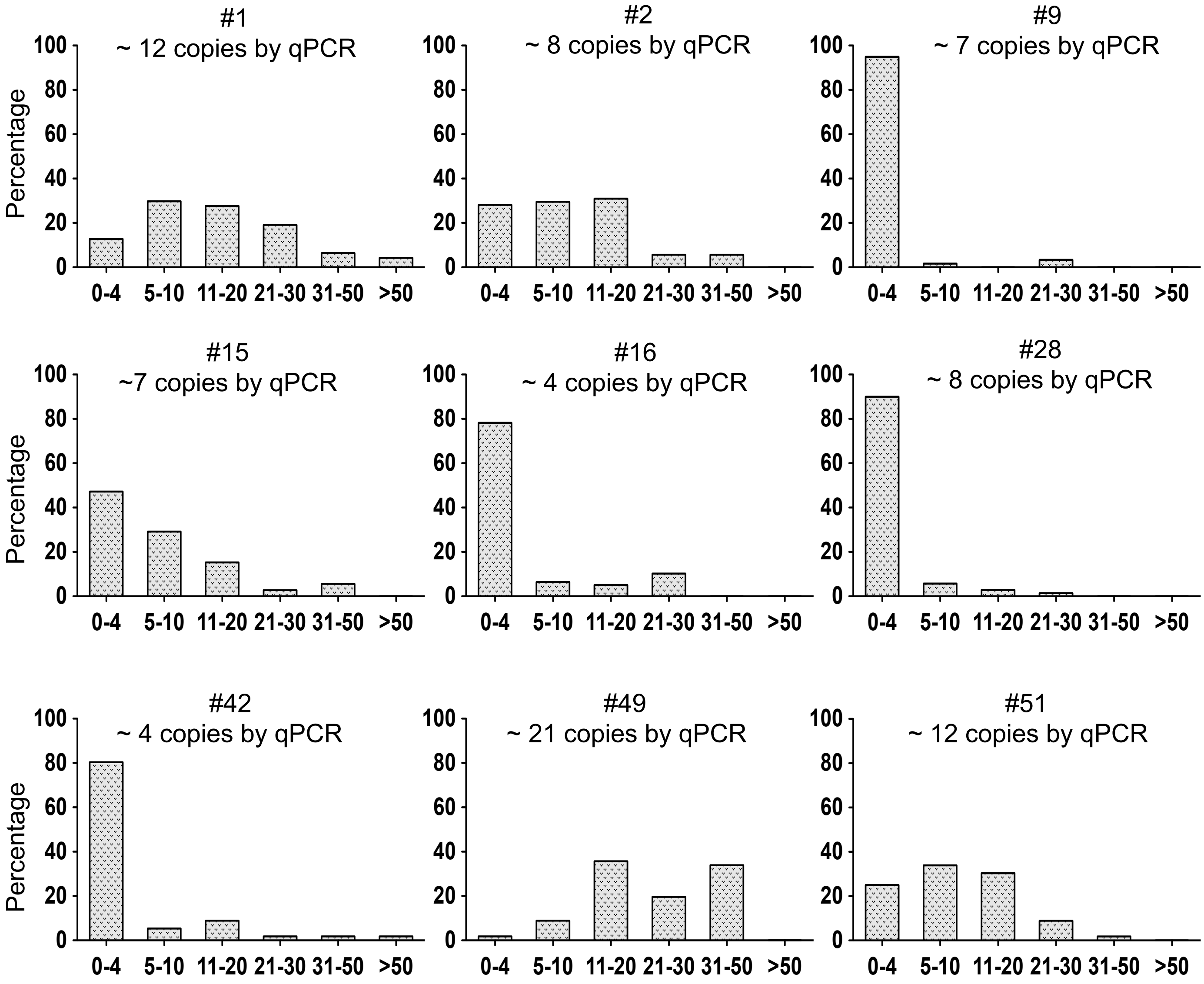

Figure S7. Distribution of DM amplification in glioma cell lines.
a) Examples of stained interphase nuclei from three different cell lines. Scale bar: 5 µm. b) Glioma cell lines with Pdgfra amplification were quantified for the number of *Pdgfra* FISH signals per nucleus. DM numbers are variable among tumor cells, consistent with a model of random distribution of DM copies to daughter cells during cell division. Average copy numbers in each cell line is listed on top of each graph.
